# Elements of the Comparative Physiology of the Domestic Mammalia

**Published:** 1838-10

**Authors:** 


Art. V.—*Lehrbuch der vergleichenden Physiologie der Haus-Saüge-thiere.* Von Dr. E. F. Gurlt, Professor an der Königl. Thierarzneischule in Berlin. *Mit drei Kupfertafeln.*

*Elements of the comparative Physiology of the Domestic Mammalia.* By Dr. E. F. Gurlt, Professor at the Royal School of Veterinary Medicine in Berlin. *With three Plates.—Berlin,* 1837. 8vo. pp. 376.

The Berlin school of veterinary medicine is probably one of the most complete in Europe. The buildings are situated in a large garden, and comprehend several lecture-rooms, the dissecting-rooms, workshops, stables, and an excellent museum of comparative anatomy and physiology. We believe there are at least eight teachers connected with this institution. Dr. Gurlt is professor of anatomy, and, with two assistants, superintends the dissecting-rooms; Dr. Erdman is professor of chemistry; Dr. Albers, of physiology and pathology; Hertwig lectures on the practice of medicine, and has a regular *clinique* on the diseases of animals. There are, besides, lectures on botany and natural history; and a journal, embracing all the departments of veterinary medicine, is edited by Gurlt and Hertwig. The facilities afforded by this institution, we are persuaded, have contributed materially to cherish the spirit of experimental research which distinguishes the Berlin school of medicine.

Dr. Gurlt's work affords ample evidence that he has availed himself of his opportunities: it contains a luminous compendium of physiology, with all its recent improvements; and many of the subjects are enriched by interesting original observations. The work is divided into two parts, General Physiology and Special Physiology: in the first of these the author treats successively of the *natural history* of the domestic mammalia, the *microscopic anatomy* of the tissues, *chemistry* and *physics* in their application to physiology, and concludes with a general survey of the phenomena and conditions of *life*. The domestic mammalia he refers to four orders: the horse and ass to the Solidungula; the ox, sheep, and goat to the Ruminantia; the dog and cat to the Carnivora; and the sow to the Pachydermata. Dr. Gurlt thinks it more advantageous to retain the Solidungula as a distinct order, than to include them in the Pachydermata, as is done by Cuvier.

The Anatomy of the Tissues contains all the late discoveries on this subject. The descriptions are short, distinct, and well illustrated by engravings, which we find, on the whole, to be very correct representations of the microscopic appearances. The structure of cartilage, bone, and teeth is very fully explained in conformity to the latest observations of Purkinje and Müller.

The medullary nervous tissue is described in accordance with the discoveries of Ehrenberg, as noticed in Art. VIII. of our present Number; but Dr. Gurlt agrees with those who believe that the beaded appearance presented by the smaller fibres is owing to a change which they gradually undergo after death. He uniformly found the fibres cylindrical in the valve of Vieusseus in the horse, if examined in its natural state, immediately after death; but, when kept for some time, they presented the varicose dilatations.

Special Physiology is arranged under four subdivisions: 1, the vital organs and functions; 2, the generative organs and functions; 3, motions; 4, the sentient organs and functions, including the mental manifestations.

The sudorific glands, first discovered in the human skin by Purkinje and Breschet, have been very carefully examined by Gurlt in the domestic mammalia. He gives a short account of their structure, in the Anatomy of the Tissues; but, in treating of the organs of secretion, he gives a fuller view of their peculiarities in different animals.

"The matter of perspiration is secreted from sudorific glands, very recently discovered. In the *horse* they are found in all parts of the skin, and consist of convoluted bags. Those situated in the skin of the organs of generation are the largest, and are of a brownish colour, owing to a brownish granular matter contained in them: in all other situations they are more free from colour, and more transparent. On the bare skin under the tail they are rather larger than in any of the parts covered by hair. The ducts are uniformly straight, or but slightly serpentine. In the *ox*, the sudorific glands, though not less numerous, are of much smaller size than in the horse, and consist of simple sacs of an oval shape, with ducts which are bent, here and there, in their course. In the *sheep*, the glands are about the same size as those of the hairy parts of the skin of the horse, and each consists of a convoluted bag, gradually passing into a duct with many serpentine bends. In the *sow*, also, the glands are of the same size and structure as in the hairy parts of the skin of the horse. The duct issues from the gland abruptly, and makes few bends. In the *dog* and *cat*, it is difficult to discover the sudorific glands: they are of minute size. Each consists of a simple oblong sac, and the duct proceeds in single bendings to the surface of the skin. On the skin of the nose, and on the soles of the feet, the glands are larger than in other parts, and consist of convoluted sacs." (p. 193.)

The meibomian glands are represented as situated *exteriorly* to the cartilages of the eyelids, between the cartilages and the orbicularis palpebrarum. We were surprised to meet with this inaccuracy in an author who, in preparing his interesting paper on the hair follicles and sebaceous follicles, must have examined the roots of the cilia and the adjacent structures. The meibomian glands are situated in grooves on the *inner* surface of the cartilage, or, according to Zeiss, in channels in the substance of the cartilage. We have not been able to distinguish the thin film of cartilage described by Zeiss, but there is no doubt that the glands are nearer to the inner than to the outer surface.

The second subdivision of the Special Physiology is on Generation and Development. It is very full, contains several original observations, and will be interesting to every student of physiology. Some parts might be made more intelligible by a few additional engravings. The exochorion and its villi are represented as originating in the vitellary membrane of the ovum. This view of the origin of the chorion, first proposed by Baer, has been very generally adopted by subsequent enquirers; but it follows, from the researches of Mr. Wharton Jones on the ova of rabbits, that the vitellary membrane and its contents, at a very early period, acquire a gelatinous envelope, which is the true rudiment of the exochorion; while the vitellary membrane gradually disappears. (See *Philosophical Transactions* for 1837, p. 339.)

The nervous matter of the brain and spinal marrow is described as a *deposit* or *precipitate* ("ablagerung") from the *fluid* contained in the dorsal canal. A similar origin is ascribed to the retina, (pp. 253, 255, 257.) The only ground alleged in support of this hypothesis is, that the fluid, at first transparent, afterwards becomes turbid; but we cannot assent to a view so inconsistent with the general analogies of development on such slender evidence.

There is no notice of the membranous vestibule and semicircular tubes which float in the liquor Cotunnii of the labyrinth.

The vitreous body of the eye is erroneously described as slightly *increasing the divergency* of the incident rays of light, because its *refractive power is less* than that of the lens. This is a mistake which has frequently been made by physiological writers. Rays passing from a denser into a rarer medium always become more *convergent* when the surface of the denser medium is convex, and the surface of the *rarer medium concave,* as is the case with the limiting surface of the lens and vitreous body.

The microscopic structure of some of the organs is described in the anatomy of the tissues; of others, with the function of the organ, or under the head of Nutrition or the head of Development. In some cases the structure of the same organ is described under two or three different heads: this tends to perplex the reader, and constitutes the chief defect in the plan of the work, which in all other respects is very clearly arranged.

